# Association Work

**Published:** 1886-01

**Authors:** J. Taft


### THE DENTAL REGISTER.

VOL. XL.] JANUARY, 1886. [No. 1.

### COMMUNICATIONS.

### Association Work.

Read before the Ohio State Dental Society.

BY J. TAFT.

The first Dental Society in this country, the American Society of Dental Surgeons, was organized in 1841, forty-four years ago. The total membership of all societies in the country, is 4,280, this includes both active and honorary members. This number may be reduced at least one-third, because of persons having membership in one or more societies, making the estimated number, 2,850. Take from this number, the honorary members, which is about 300, and it leaves the active membership of all the societies, about 2,550; thus, not over one in five, in the entire profession, is in any wise connected with association work —and if from the above number, is taken the names of those members who do not actively engage in the work, the number would probably be reduced below one thousand; thus, not over one in twelve or fourteen, labor in this direction for the profession. It has generally been the aim, in our societies, to present by papers and discussion, various practical questions of the profession, those pertaining to methods and modes. Not only in public discussion, but in special conference, much has been accomplished in the way of making the particular attainments of individuals a common inheritance, and in this way, not only does benefit accrue to others, but the giver, by the very contact, receives new stimulus, and oftentimes new thought and ideas.

Not only by this means has knowledge been increased and extended, but methods of demonstration by Clinics has been utilized, to the benefit of multitudes in and outside of the profession. For the introduction and practical use of this mode of instruction in Dental Societies, the profession is chiefly indebted to Dr. W. H. Atkinson, who, with a prophetic eye, in 1859, forecast the possibilities of associated work in our department of the healing art. In Associations, men being brought together, have learned the strong points of their fellows, and the weak points, each of himself, these lessons are always valuable. The strength and attainments of others may by right means become our own, and we in turn, may minister to others, and at the same time our own feebleness be converted into strength and power. The Societies have done much in molding the character of the profession, and in elevating its status; this influence has been operative not only upon those actively engaged, but it has made a marked impression on those who contemplate entering its ranks. The time has passed, when a young man can enter an office, and by agreement, complete his course of instruction in from three to twelve months and go out with an endorsement by his preceptor, as entirely competent to discharge the varied and difficult duties of the Dental Surgeon. The power of associative influence has made a marked impression on our special Institutions of learning; as our associations have from time to time taken higher ground and advanced positions, our colleges have been compelled to recognize the fact and modify their plans and methods accordingly.

The possibility of regulating the practice of dentistry by legal enactments, was until a comparatively recent period, entertained by but few, but as it came to be discussed and studied, it received more favorable consideration, until now almost every State in the Union, at least that has a State Society, has a law regulating the practice of dentistry, and one of our Territories also has a Society and a law similar to those of the States. Our Societies have also exercised a healthy stimulus upon our literature; many have thus been induced to write, who otherwise would have done little or nothing in this direction.

That association effort has done much for the development and up-building of the dental art and science, none will deny, indeed there would have been but little progress compared with that made, without such organized work.

However pleasant and gratifying it may be to review the work accomplished in the past, it will not be without profit to forecast the tendency of the future, and to suggest, and even plan methods that seemingly might be easily carried out.

While with Institutions, as with individuals, there is a disposing power that rules over the plans and devices of man, yet, we are fully warranted in making plans and devising methods of work which will always be sure of success in proportion as they may be in harmony with that over-ruling Power. I now propose to note a few points in which association work might be improved. No criticism need here be indulged in, upon the conduct and work of these organizations, let us rather look upon the bright side and make the most of what has been accomplished, and seek for more and better things in the future. All will concur in the suggestion that each and every State should have its Dental Society, and even each Territory having ten or more practitioners who could conveniently meet. Every district having ten or more reputable dentists, should have its organization embracing towns within convenient distances. The Society of each State should meet annually, those of the cities, monthly, and those of the smaller towns and districts, as often as convenience would warrant, perhaps from two to six times a year. There are four classes of societies; the American or National, the State, City and District. The object is usually stated in the organic law of each, to be, to cultivate good-fellowship, and promote the interests of our art and science. The work that may be done by each of these classes will necessarily differ in some respects, in others, there will be work common to all. The American Association is a delegated body whose members come from all other organizations of our profession in the country, so far as they wish to be represented, and this is almost universal. This is not a legislative body, further than its own action is concerned, notwithstanding it has an interest in, and should exercise a wholesome corrective and stimulative influence upon all other societies, particularly those represented.

This fostering care was exercised more fully in the early periods of the society than in later years, it seemed then to recognize much more fully, the importance of this work, than now. For several years it had a committee whose duty it was to aid in the organization of local societies, and to do whatever was practicable in the way of aiding those already organized. A report was made by that committee on the state of the Association and the condition of the profession, generally. While the presentation and the discussion of modes of practice, and the details per taining to it are interesting and valuable, would it not be better that this body give more attention to the general interests of the profession, than it has been disposed to do for some years past. The great moving and controlling influences that are operative in the profession, should certainly come within the perview of this body, and though it is not invested with legislative power, yet, in an advisory way, it may serve the interest of the profession in a very marked degree; in some respects directly, and in others indirectly, but resulting in great good. The Dental Profession of Great Britain is outstripping that of this country in some respects; there, the law regulating the practice, involving registration and all the details of its requirements, is a unit, uniform in action and results. There, the profession is brought to a uniform standard of requirements, registration is universal; violators of the law are punished. In this country perhaps it is not possible to attain the same uniformity in respect to requirements and law, as in England. Every State enacts its own law, and adopts such requirements as it may see fit, and many of those laws are defective. May not the American Association exercise a wholesome influence over the various State Societies here represented, in bringing about a more uniform standard of requirements, in making registration requisite in every State. It certainly is within the province of this body, to labor with the end in view, not only of elevating the standard of professional attainments, but in producing uniformity in all the great interests of the profession, so far as practicable. Declarations have here been made in reference to our modes of education, which has made decided impression, and improvement, in some respects. All Societies and Institutions here represented, should feel entirely at liberty to ask advice and direction in all matters where they might regard advice as valuable.

The time and work of our State Society should be given, more to the broad interests of our profession, and relate less to the matters of practical detail. The State Societies can well afford to foster those of more local character; every State should look well to the execution of its State Law. There are also certain aspects of the educational problem which they may well consider, and indeed, act upon. It is proper and desirable that State Societies, and all local Societies should consider well, the subject of pupilage. The qualifications and character of those who are now entering, and are hereafter to enter our profession, is a very important question, and one which those Societies can reach in a very efficient manner. They can decide as to the qualifications of those whom the members will encourge to become students. No one, now-a-days, who thinks of entering the profession, will attempt to begin a course without consulting some one whom he regards as competent to give advice, and in a majority of instances such persons interested, are members of some Dental Society. By establishing a standard, and adhering to it, great good would be accomplished. It has already been shown that a very small proportion of the profession is embraced in our Dental Societies. Laboring to build up the membership in every one, would be productive of great good. Instead of there being but 2,500 members of the profession in our Societies, there should be from eight to ten thousand, and there would be, if all those in our Societies would go actively to work with a view of building up in this respect. Two-thirds to three-fourths of all our Societies could be instrumental each year, in bringing in from one to three new members, by well directed effort. Why shall this not be done? Every State Society should become incorporated. To become an incorporated body is a very easy matter; for this, the common law makes ample provision. Another direction in which effort may be made, and with great profit too, is where a Society is held in one locality, the establishment of a library, apparatus, etc. Such an establishment would constitute a nucleus that would involve permancy and stability, and would be of immense value and benefit in many respects. In such a Museum, specimens and appliances would find a safe place of deposit, where they would not only not be lost or destroyed, but would be utilized for information of all the members. Apparatus for illustration could be accumulated, but more particularly the collection of a library would prove valuable. Very few in our profession have access to more than a small number of our standard works. The establishment of a Museum and Library would be an element of strength as well as valuable in other respects.

There are really four classes of Societies; the National, the State, the District and the City, each having some distinctive work. The chief value of the National Society is in the unifying influence which it should exercise over all bodies represented in it, and in fostering association work generally.

In regard to State Societies, the question of a fixed habitation is one well worthy of consideration.

City, or mere local bodies, will find much to occupy the attention that would not be practicable in those of a more migratory character; thus it would appear that each class of our Associations has a distinctive form of work, to which special attention should be given, and which if well done, would yield greater results than have yet been realized.

